# Association between body fat and bone mineral density in non-obese post-menopausal women over 60 years old

**DOI:** 10.22088/cjim.12.2.200

**Published:** 2021-03

**Authors:** Seyed Amirhossein Hosseini, Seyed Reza Hosseini, Reza Ghadimi, Hajighorban Noreddini, Ali Bijani

**Affiliations:** 1 Social Determinants of Health Research Center, Health Research Institute, Babol University of Medical Sciences, Babol, Iran

**Keywords:** Bone mineral density, Body fat, Post-menopausal, Women

## Abstract

**Background:**

Loss of bone mineral density is one of the most important complications of menopause. The results of studies about the relation between body fat and bone mineral density are controversial. The aim of this study was to determine the association between fat mass and bone mineral density on non-obese post-menopausal elderly women.

**Methods:**

This cross-sectional study is a part of the second phase of the Amirkola Health and Ageing Project (AHAP) that has been done on 356 elderly women with BMI of 18.5-30. Bone mineral density (BMD) and total body fat were measured using the Hologic Horizon-WI densitometer. Statistical tests were ANOVA, Pearson’s correlation coefficient and multiple linear regression and a p- value less than 0.05 was considered significant.

**Results:**

The mean age of the participants was 70.22±7.34 and the mean age of menopause was 47.68±5.05. Women with highest fat mass had a greater spine, femur and whole-body BMD (p<0.0001). In this study, we observed a direct and positive significant correlation between body fat mass and BMD at spine (r=0.308), femur (r=0.420) and whole body (r=0.312) (p<0.0001). Adjusted linear regression showed positive effect of fat mass on BMD on all three anatomical sites especially in total femur (β=0.254, p<0.0001).

**Conclusion:**

This study showed a positive correlation between fat mass and bone mineral density at all sites in post-menopausal women.

Osteoporosis is a common chronic disease around the world with higher prevalence among elderly and menopausal women (1, 2). The prevalence rate of osteoporosis among Iranian menopausal women have been reported 32% (26-39%) (3). Osteoporosis is characterized by loss of bone mineral density and deterioration of bone tissue leading to bone fractures (4). Female sex hormones have many positive effects on well-being and healthiness of women throughout their life. Women experience a great number of changes in their hormone levels during menopause including an increase in FSH and decrease level of estradiol which both happen to various degrees in different individuals. The hormonal changes start years before the last period and because menopause in most women occurs from age of 46 to 52, we can conclude that women are in menopausal state for almost one-third of their lifetime (5). Loss of bone mineral density is one of the most important complications of menopause which often is the result of decrease in ovarian hormones, insufficient calcium, phosphorus and vitamin D intake, smoking, taking corticosteroid drugs and some endocrine diseases that cause hormonal imbalance like hypothyroidism and hyperparathyroidism (6, 7). 

Ageing, metabolic factors, low levels of hormones due to menopause accompanied by low physical activity could be a cause of overweight and a reason for a higher fat mass (8). The role of sex hormones specially estrogen in maintaining bone content is well established. The deficit in estrogen results in bone loss through an increase in bone turn over and decrease in bone formation so, in the first 5 to 10 years of menopause, 25 to 30% of trabecular bones and 10-15% of cortical bones are lost (9). Body composition which consists of bone, lean mass and fat mass that is accountable for 90 to 95 percent of body weight, has a significant effect on BMD and bone mineral content (BMC) (10). Many studies have been conducted to determine the relationship of body fat tissue and bone mineral density but the results from these studies have been controversial. Some of these studies showed positive correlation of fat mass on bone mineral density (11-15). Other studies have shown fat mass either had no effect on spine and femur BMD (4) or had negative effect and was associated with the increased risk of bone fractures (16-19). Therefore, because of the inconclusive and controversial results from previous studies, the current population-based study has been designed to investigate the relationship between fat mass and bone mineral density in non-obese (BMI<30) post-menopausal elderly women who are over 60, in the city of Amirkola, northern of Iran.

## Methods

This cross-sectional population-based study is a part of the second phase of the Amirkola Health and Ageing Project which is an ongoing cohort project that started since 2011 on elderly residents of Amirkola city, northern Iran (20). This project was approved by the Research Ethics Committee of Babol University of Medical Sciences (Ethical registration code: IR.MUBABOL.HRI.REC.1398.323). Study population for this research are elderly women aged 60 and over. A random sample of 356 elderly women with BMI of 18.5-30 were selected. By considering the 95% confidence level and 80% power and assuming a correlation coefficient equal to 0.15 for the relationship between body fat and bone mass, the sample size was estimated to be about 350 people. The exclusion criteria include presence or history of any cancer, history of vertebral or femur fractures, losing weight 7 kilograms or more within the last 6 months and the menopausal age of less than 35 years. The demographic data including age and menopausal age were acquired through questionnaires. Anthropometric data including height, weight, and waist circumference were measured and the BMI was calculated. Height (cm) was measured with stadiometer with 0.1 cm accuracy and weight (kg) was measured with a digital Seca scale while wearing minimum clothes and no shoes with 0.1 kg accuracy.

Bone mineral density of spine and femur and whole body has been measured using a Hologic Horizon-WI densitometer. BMD was measured in grams per square centimeter (g/cm2) using dual energy x-ray absorptiometry in lumbar spine (L1–L4), femoral neck, total hip, and whole body. Fat mass in grams (g), consisting visceral adipose tissue (VAT), local and total body fat measured using the same Hologic Horizon densitometer. 

Percentage of body fat was obtained by dividing the total fat mass by the total body weight and multiplying by 100. Participants have been classified into three groups based on FM. Group 1 consisted of participants with less than 23650 grams of FM, second group with 23651-28100 and the third group was participants with 28100 grams of fat or more. 

Physical activity was collected using the Physical Activity Scale for the Elderly (PASE) questionnaire and through interview with elderly people (21). The questionnaire has three parts including leisure time, household and work-related activities. The total physical activity score for each individual was calculated as between 0 and 400; higher scores means higher physical activity levels.

Then the results have been analyzed using ANOVA, Pearson’s correlation coefficient and multiple linear regression tests. A p- value equal or less than 0.05 was considered statistically significant. 

## Results

**Figure 1:**
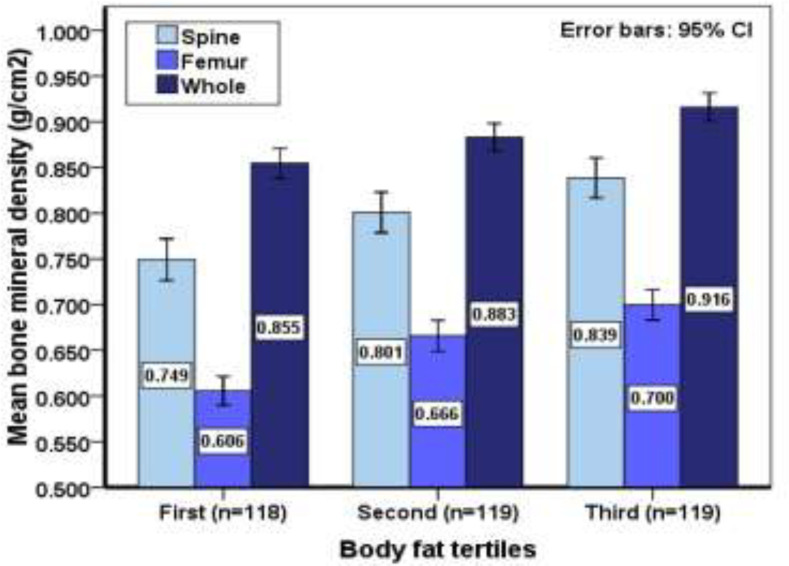
Association between BMD and body fat in post-menopausal women in Amirkola, Iran

**Figure 2:**
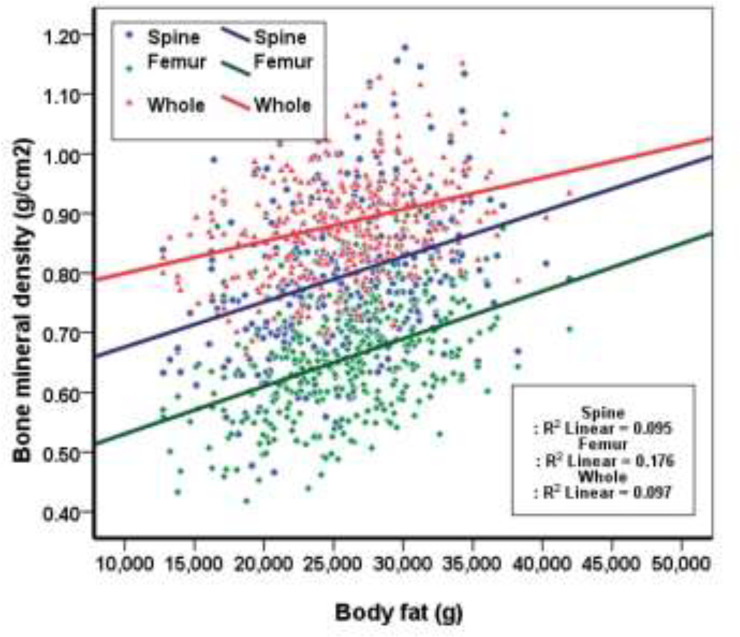
Correlation between body fat and bone mineral density in elderly women of Amirkola, Iran (N=356)

This study was conducted on 365 elderly women with a BMI of 18.5 to 30. The mean age of participants was 70.22±7.34, the mean age of menopause was 47.68±5.05, the mean waist circumference was 86.53±8.24, the mean FM was 25880.99±5183.53 and the mean BMD was 0.88472±0.08892 (table 1). As it has been shown in table 2, participants who were classified in the third group of fat mass had a greater height, weight, waist circumference and BMI in comparison to the other two groups though they had lower mean age (p<0.0001). Moreover, participants who were classified in the third group of fat mass, had a greater spine, femur and whole-body BMD (p<0.0001), this shows that with an increase in fat mass, we see an increase in bone mineral density at all sites. As it has been demonstrated in the figure 1, an increase in FM leads to an increase of BMD in all three measured sites, although this increase was greater in spine and the whole body. In this study, we observed a direct and positive significant correlation between body fat and BMD at spine (r=0.308), femur (r=0.420) and whole body (r=0.312) (p<0.0001) (table 4). Table 5 demonstrates that there is a reversed but significant correlation between age with BMD, LM and FM. This means with aging, there is a decrease in BMD, FM and LM (p<0.0001). Figure 2 shows a positive and significant correlation between FM and BMD and with increasing fat mass, bone mineral density increases. In multiple regression analysis, after adjusting for age, menopausal duration, physical activity and lean mass, there was still positive association between fat mass and BMD in all three anatomical sites, however, association was a stronger in the femoral region than other sites (table 6).

**Table1:** Baseline characteristic and anthropometric indices of elderly women of Amirkola, Iran (N=356).

	**Mean±SD**	**Minimum**	**Maximum**
Age (yr)	70.22±7.34	60	93
Menopausal age (yr)	47.68±5.05	35	60
Height(cm)	150.65±6.02	133.5	171.5
Weight(kg)	59.52±7.94	38.5	80
BMI(kg/m^2^)	26.17±2.69	18.86	29.97
Waist (cm)	86.53±8.24	64	119
Fat (g)	25880.99±5183.52	12735	41944
Lean (g)	31786.23±3503.02	22763.4	42134.9
BMD. Spine (g/cm^2^)	0.79638±0.12746	0.466	1.305
BMD. Femur (g/cm^2^)	0.65717±0.09835	0.418	1.066
BMD. Whole (g/cm^2^)	0.88472±0.08892	0.664	1.262

**Table 2:** Relationship between age, age of menopause and anthropometric indices with fat mass tertile in elderly women of Amirkola, Iran (N=356).

**Variables**	**Fat group1 (n=118)** **Mean ± SD**	**Fat group2 (n=119)** **Mean ± SD**	**Fat group3 (n=119)** **Mean ± SD**	**P-value**
Age (yr)	72.86±8.01	70.01±6.96	67.81±6.10	0.000
Age of menopause (yr)	47.54±5.12	48.32±4.72	47.19±5.26	0.208
Height (cm)	147.26±5.81	150.66±4.93	153.99±5.32	0.000
Weight (kg)	51.42±5.25	59.80±3.92	67.29±4.54	0.000
Waist (cm)	80.16±6.56	87.34±5.79	92.04±7.50	0.000
BMI (kg/m^2^)	23.74±2.32	26.38±1.89	28.38±1.40	0.000

**Table 3:** Relationship between fat mass tertile and BMD, LM in elderly women of Amirkola, Iran (N=356).

**Variables**	**Fat group1 (n=118)** **Mean ± SD**	**Fat group2 (n=119)** **Mean ± SD**	**Fat group3 (n=119)** **Mean ± SD**	**P-value**
BMD.Spine g/cm^2^)	.74924±.1247	.80087±.1217	.83862±.1208	0.000
BMD.Femur (g/cm^2^)	.60561±.0869	.66582±.0930	.69966±.0915	0.000
BMD.Whole (g/cm^2^)	.85467±.0883	88318±.0832	.91605±0850	0.000
Fat (g)	20171.750±2714.36	25941.467±1289.33	31481.792±2762.75	0.000
Lean. Fat	1.4938±.2466	1.2331±.1273	1.0795±.1188	0.000
Lean (g)	29627.554±3216.04	31926.969±3055.93	33786.028±2934.38	0.000

**Table 4:** Pearson Correlation of BMD and body composition parameters in elderly women of Amirkola, Iran (N=356).

	**BMD.Spine**	**BMD.Femur**	**BMD.Whole**	**Fat**	**Lean**	**BMI**	**Lean.Fat**
BMD.Spine	1						
BMD.Femur	0.610 0.000	1					
BMD.Whole	0.780 0.000	0.625 0.000	1				
Fat	0.308 0.000	0.420 0.000	0.312 0.000	1			
Lean	0.317 0.000	0.409 0.000	0.316 0.000	0.513 0.000	1		
BMI	0.282 0.000	0.384 0.000	0.246 0.000	0.755 0.000	0.561 0.000	1	
Lean.Fat	-0.165 0.002	-0.254 0.000	-0.167 0.002	-0.821 0.000	0.002 .975	-0.564 0.000	1

**Table 5:** Pearson correlation of age, menopausal age, anthropometric indices and BMD in elderly women of Amirkola, Iran (N=356).

	**age**	**Menopausal age**	**Height**	**Weight**	**Waist**
BMD.Spine	-0.235 0.000	0.118 0.026	0.249 0.000	0.366 0.000	0.211 0.000
BMD.Femur	-0.309 0.000	0.068 0.20	0.301 0.000	0.478 0.000	0.256 0.000
BMD.Whole	-0.367 0.000	0.092 0.083	0.300 0.000	0.366 0.000	0.150 0.004
Fat	-0.302 0.000	-0.045 0.402	0.504 0.000	0.885 0.000	0.636 0.000
Lean	-0.298 0.000	-0.012 0.823	0.593 0.000	0.788 0.000	0.469 0.000
BMI	-0.218 0.000	-0.049 0.355	0.050 0.346	0.800 0.000	0.650 0.000
Lean.Fat	0.179 0.001	0.028 0.399	-0.193 0.000	-0.546 0.000	-0.474 0.000

**Table 6.** Multiple Regression Analysis of BMD and Fat mass in elderly women of Amirkola, Iran (N=356). (Adjusted for Age, menopause duration, physical activity and lean mass)

	**Whole BMD**		**Spine BMD**		**Femur BMD**	
**Variables**	β	P value	β	P value	β	P value
Age	-0.074	0.386	0.082	0.354	-0.014	0.868
Menopause duration	-0.201	0.014	-0.233	0.006	-0.158	<0.0001
Physical activity	0.106	0.037	0.051	0.334	0.060	<0.0001
Lean mass	0.157	0.005	0.188	0.001	0.230	0.226
Fat mass	0.150	0.008	0.176	0.003	0.254	0.046

## Discussion

Identifying the factors affecting bone mineral density and modifying them are very important and vital in the prevention and treatment of osteoporosis. The most important factor of BMD is the body composition which is determined by ageing, physical activity, nutrition, menopausal state and diseases. In the present study, we investigated the relationship between fat mass and BMD in post-menopausal women. The results of this study showed that in post-menopausal women, BMD of lumbar spine, femur and whole body had a positive relationship with FM and this was significant after controlling age, menopausal duration, physical activity and LM. In this study, the participants based on fat mass have been categorized in three groups, the group with the most FM (3^rd^ group) had a higher BMD in all three regions. Other results from this study include the direct and significant correlation of FM and BMD in femur, spine and whole body, although the effect of FM on BMD was greater in femur than other sites and this was significant in multiple regression analysis after adjusting for other variables. 

Generally it has been said that higher BMI leads to higher BMD due its mechanical effect in addition to the adipocytes are an important source of producing estrogen in post-menopausal women. Estrogen is an inhibitor of bone resorption caused by osteoclasts. It seems an increase in adipose tissue in post-menopausal women causes suppression of osteoclasts activities, thus it increases bone mineral density (22). There is an established relationship between obesity and insulin resistance, obesity can also have a role in other abnormalities like excess production of androgens and estrogen from ovaries and a decrease in production of sex hormone binding globulin (SHBG) from liver. These changes due to an increase of fat mass can lead to higher BMD because of inhibition of osteoclasts’ activities and possibly an increase in osteoblasts’ activities (23). The results of some studies were similar to our study, for example the studies by Namwongprom et al. (14) in Thailand, Ilesnami-Oyelere et al .(24) in New Zealand, Marwaha et al. (25) in India, Marin-Mio et al. (26) and Xin Shi et al. (27) showed a positive relation between fat mass and bone mineral density. Meanwhile, other studies had different results. Studies by Salamat et al. in Iran (28) , Kim et al. in South Korea (16), Liu et al. (29) and Alishiri et al. in Iran (30) showed a negative relationship between fat mass and BMD.

The reason for controversy between the results of all mentioned studies could be due to the differences in the populations, age group, menopause status, design of the study, methods of sampling, sample size, genetics and or differences in races. The strengths of this study were using data from a population-based cohort study in Amirkola, large sample size and measuring BMD in three different anatomical sites, measuring LM and categorizing samples-based on the fat percentage and comparing them. The first limitation of this study was cross-sectional design of the study, which makes it hard to find the cause-and-effect relationship. The other limitation of this study was the lack of control of other factors effecting on BMD such as lifestyle, nutrition, calcium intake, smoking and measuring serum levels of sex hormones. The third limitation of this study was the inaccuracy of menopausal age. 

The findings of this study showed a positive correlation between fat mass and bone mineral density in all three anatomical sites especially in femur in post-menopausal women. This relationship remained after the adjustment for age and lean mass. It seems that being overweight for post-menopausal women, may have beneficial effect on bone health. 
